# *Trichoderma pubescens* Elicit Induced Systemic Resistance in Tomato Challenged by *Rhizoctonia solani*

**DOI:** 10.3390/jof9020167

**Published:** 2023-01-27

**Authors:** Said Behiry, Seham A. Soliman, Magdy A. Massoud, Moawad Abdelbary, Ahmed M. Kordy, Ahmed Abdelkhalek, Ahmed Heflish

**Affiliations:** 1 Agricultural Botany Department, Faculty of Agriculture (Saba Basha), Alexandria University, Alexandria 21531, Egypt; 2 Plant Protection and Biomolecular Diagnosis Department, ALCRI, City of Scientific Research and Technological Applications, New Borg El Arab City 21934, Egypt; 3 Plant Protection Department, Faculty of Agriculture (Saba Basha), Alexandria University, Alexandria 21531, Egypt

**Keywords:** *Rhizoctonia solani*, *Trichoderma pubescens*, tomato, ITS, defense, genes, enzyme, HPLC

## Abstract

*Rhizoctonia solani* causes severe diseases in many plant species, particularly root rot in tomato plants. For the first time, *Trichoderma pubescens* effectively controls *R. solani* in vitro and in vivo. *R. solani* strain R11 was identified using the ITS region (OP456527); meanwhile, *T. pubescens* strain Tp21 was characterized by the ITS region (OP456528) and two genes (*tef-1* and *rpb2*). The antagonistic dual culture method revealed that *T. pubescens* had a high activity of 76.93% in vitro. A substantial increase in root length, plant height, shoot fresh and dry, and root fresh and dry weight was indicated after applying *T. pubescens* to tomato plants in vivo. Additionally, it significantly increased the chlorophyll content and total phenolic compounds. The treatment with *T. pubescens* exhibited a low disease index (DI, 16.00%) without significant differences with Uniform^®^ fungicide at a concentration of 1 ppm (14.67%), while the *R. solani*-infected plants showed a DI of 78.67%. At 15 days after inoculation, promising increases in the relative expression levels of three defense-related genes (*PAL, CHS,* and *HQT*) were observed in all *T. pubescens* treated plants compared with the non-treated plants. Plants treated with *T. pubescens* alone showed the highest expression value, with relative transcriptional levels of *PAL, CHS*, and *HQT* that were 2.72-, 4.44-, and 3.72-fold higher in comparison with control plants, respectively. The two treatments of *T. pubescens* exhibited increasing antioxidant enzyme production (POX, SOD, PPO, and CAT), while high MDA and H_2_O_2_ levels were observed in the infected plants. The HPLC results of the leaf extract showed a fluctuation in polyphenolic compound content. *T. pubescens* application alone or for treating plant pathogen infection showed elevated phenolic acids such as chlorogenic and coumaric acids. Therefore, the ability of *T. pubescens* to inhibit the growth of *R. solani*, enhance the development of tomato plants, and induce systemic resistance supports the application of *T. pubescens* as a potential bioagent for managing root rot disease and productivity increase of crops.

## 1. Introduction

Tomato (*Lycopersicon esculentum* L.) is ranked among vegetable crops as the 2nd most consumed globally, following potato [1]. It belongs to the Solanaceae family and contains tomatine and tryptophan, which are considered nutritive and vital compounds for human health. Among processed vegetables, tomato ranks first in area used for growing globally, followed by potato. Many phytopathogens attack tomato plants, which leads to serious diseases. The diseases of tomato plants can be caused by fungi, nematodes, bacteria, and viruses [2,3]. Soil-borne fungi such as *Fusarium oxysporum*, *Rhizoctonia solani*, *Verticillium,* and *Pythium* can cause root rot diseases in tomatoes [4]. Among these, *R. solani* is the main pathogenic fungus. *R. solani* is a harmful soil-borne pathogen that is responsible for substantial crop losses all over the world [5]. One of the most serious tomato diseases, *R. solani-* root rot and damping-off, is managed using fungicides. There are various symptoms of *R. solani* infection, which is responsible for seedling death in the cultivated area [6]. Circular patches on stem seedlings are the first symptom of the disease, followed by abrasions of seedlings at the soil surface [5,6]. The tomato root rot disease caused by *R. solani* pathogen mostly occurs during the seedling and mature plant stages, leading to about 60% yield loss [7].

The most widely used method of preventing these diseases is chemical control using fungicides; however, due to physiological pathogen races becoming more resistant to fungicides, there is a limit to the chemical’s application for this purpose against fungal pathogens [8,9,10]. There are also hazards to human health and the environment related to using chemicals [11]. Biological control agents can inhibit pathogen growth using specialized mechanisms such as parasitism, antibiosis, and competition for nutrients and space in the rhizosphere zone [12]. The use of beneficial microorganisms, such as biocontrol agents, is being promoted for their potential in sustainable agriculture. *Trichoderma* species have been used to control the phytopathogenic fungi, such as *R. solani, Fusarium* spp., *Phytophthora palmivora*, *Botrytis cinerea*, and *Pythium* spp., in tomato, rice, papaya, castor beans, tobacco, and bean crops [4,5,13,14]. The effect of *Trichoderma* species as biocontrol agents is due to their rapid growth and tolerance of adverse environmental conditions [15]. *Trichoderma* species have strong antagonistic and mycoparasitic effects on plant pathogens, which allow them to reduce the plant disease severity, while hyperparasitism is considered the most critical mechanism for *Trichoderma* [16,17]. Cell-wall-degrading-enzymes (CWDEs) such as chitinases, glucanases, and proteinases can be released by *Trichoderma* spp. during the hyperparasitic phase [18]. The plant pathogen cell wall can be weakened by the secreted CWDEs [19].

During the *Trichoderma*-plant interaction, various classes of metabolites, such as proteins with enzymatic activity, low molecular weight compounds related to the fungal or plant cell wall [20], and other secondary metabolites trigger plant defense mechanisms against pathogens [21], by activating the pathogenesis-related proteins that reduce the disease symptoms. A systemic acquired resistance mechanism (SAR) is activated when a plant comes into contact with a pathogen. However, when they interact with a non-pathogen organism, the plants activat an “induced systemic resistance” (ISR) mechanism [22]. Therefore, the current study aims to evaluate the effect of *Trichoderma* on the root rot of tomato caused by *R. solani* compared with fungicides under laboratory and greenhouse conditions. Additional effects of *Trichoderma* were evaluated on growth metrics, chlorophyll content, oxidative and antioxidant enzymes, phenolic and flavonoid content, and the expression levels of genes involved in defense.

## 2. Materials and Methods

### 2.1. Isolation and Identification of Pathogen and Bioagents 

From different fields in the governorate of El-Behira, Egypt, tomato plants showing rot symptoms on roots were collected. From the collected samples, the fungal pathogen was isolated and characterized using cultural and morphological parameters, as well as ITS sequence [23]. *Trichoderma*-specific-medium (TSM, 0.2 g of MgSO_4_∙7H_2_O, 0.9 g of K_2_HPO_4_, 0.15 g of KCl, 1.0 g of NH_4_NO_3_, 3.0 g of glucose, 0.15 g of rose Bengal, 20 g of agar, 0.25 g of chloramphenicol, 0.3 g of p-dimethylaminobenzenediazo sodium sulfonate and 0.2 g of pentachloronitrobenzene/L of distilled water) was used for the isolation of *Trichoderma* from rhizospheric samples of soil collected from different fields. The technique of serial dilution was used to isolate the antagonistic *Trichoderma* spp. Dilution of 1 × 10^−3^ (1 mL) was poured onto Petri plates containing TSM as used by Elad et al. [24]. Using a hyphal tip technique, the acquired cultures of *Rhizoctonia solani* fungi were purified and kept on slants of potato dextrose agar (PDA, filtrate of boiled 200 g potato, 20 g dextrose, and 20 g agar; all the ingredients were mixed and made up to one litre of distilled water) for subsequent procedures. 

### 2.2. Effect of Trichoderma Isolates on Growth of Rhizoctonia

A dual culture technique was used to evaluate the efficacy of *Trichoderma* isolates in inhibiting *R. solani,* as described by Fahmi et al. [25]. Seven-day-old cultures of *Trichoderma* isolates and *R. solani* (R4) were used in this test. PDA Petri plates were inoculated with a 5 mm disc of *Trichoderma* positioned diametrically opposite to the *R. solani* disc. After five days of incubation at 25 ± 2 °C, *R. solani* radial growth was recorded. Petri plates with an agar disc (5 mm) were used as the control instead of *Trichoderma*. These tests were conducted in triplicate for all treatments. From the recorded radial growth of *R. solani* (mm), the inhibition percentage was estimated as follows:

$$\mathrm{Inhibition}\;\%=\left(\frac{C-T}{C}\right)\times 100$$

where C = *R. solani* growth in control, T = *R. solani* growth in treatments.

After 4 days, a light microscope was used to investigate the mycoparasitic interaction zone between *Trichoderma* and *R. solani* on PDA.

### 2.3. Effect of Fungicides on R. solani In Vitro

Using a poisoned food technique [26], different fungicides were tested for their ability to prevent the growth of the *R. solani* isolate. Three fungicides were used: Uniform^®^ (active ingredient: Azoxystrobin 28.2% (*w*/*w*) and Mefenoxam 10.8% (*w*/*w*), Syngenta, Wilmington, Delaware, USA), Rizolex^®^ (active ingredient: Tolclofos-methyl 50 % (*w*/*w*) WP, Sumitomo Chemical Corp., Nihonbashi, Chuo-ku, Tokyo, Japan), and Hattrick^®^ (active ingredient: Tebuconazole 6% FS, Shoura chemicals, Egypt). PDA media plates were incorporated with fungicides at a final concentration of 1 ppm. The mycelial disc of the *R. solani* (5 mm) from the 7-day-old culture was placed in the middle of the Petri plate and incubated at 25 °C. PDA without fungicide was used as a control. Three replicates were used for each treatment, and the experiment was arranged in a completely randomized design (CRD). The radial growth of *R. solani* was recorded after 7 days to calculate the inhibition percentage.

### 2.4. Identification of R. solani and Trichoderma Isolates

Based on morphological characteristics and molecular testing using the ITS, *rpb2*, and *tef*-1 genes, *R. solani* and *Trichoderma* isolates are identified [27,28,29]. Primer sequences used to identify *R. solani* and the most effective *Trichoderma* isolate are presented in Table 1. From isolated fungi, 0.5 g of fresh 5-day-old mycelium was taken by a sterile scalpel for DNA extraction. Using 1 mL of CTAB extraction buffer, the mycelium of fungi was crushed in a mortar and pestle before being heated at 65 °C for 30 min in a water bath. Chloroform/isoamyl alcohol was then mixed with the sample in equal volume and gently mixed for 30 min, then centrifuged for 10 min at 10,000 rpm. Chilled isopropanol (600 µL) was added to the obtained supernatant and kept for 2 h at 4 °C. To precipitate, the DNA samples were centrifuged for 10 min at 10,000 rpm. Ethanol (70%) was used to wash the collected pellet, then left for 4 h to get air dried to remove any remaining ethanol.

PCR reactions with DNA template (1 µL), 10 pmol of forward and reverse primer (0.5 µL of each), and 10 µL of 2× Taq-Ready-Mix (Enzynomics Inc., Daejeon, Republic of Korea), the reaction volume adjusted to 25 µL using Milli-Q water. A Techne Prime Thermal Cycler (Cole-Parmer, Staffordshire, UK) was used for the cycling, which was carried out as follows: a 95 °C for 4 min initial denaturation, then 35 cycles (94 °C for 45 s, 55 °C for 45 s, and 72 °C for 1 min) and 72 °C for 5 min as a final extension [5]. MEGA X software was used to align the nucleotide sequences after the PCR amplification product was sequenced. The acquired sequences were compared to the database in GenBank using the GenBank BLAST program.

### 2.5. Biocontrol Potential and Plant Growth-Promoting Abilities of Trichoderma Isolate against R. solani on Tomato Plants

The most effective *Trichoderma* isolate in inhibiting *R. solani* R4 under lab conditions was evaluated under greenhouse conditions (28 ± 2 °C temperature, humidity 80–90%, and day/night 12 h photoperoid) based on a pot experiment for their effect on *R. solani* R4 and plant growth. Sterile soil was used to fill plastic pots (20 cm) and pre-inoculated with *R. solani*; four weeks of tomato (var. Peto 86) seedlings were transplanted. Seedling roots were dipped for 2 h before transplanting in the inoculum of *Trichoderma* (1 × 10^8^ spores/mL) [24]. Pre-autoclaved wetted barley grains (500 g) were inoculated with 2 discs of *R. solani* fungus (5 mm in diameter) and incubated for 7 days at 25 ± 2 °C. After incubation, the fungus-grain inoculum was air-dried, blended to a powder form, and added to the pots 48 h before transplanting (10 g inoculum/kg) where the inoculum was initiated nearby to the root pan and crown of the plant. The fungicide was applied to the soil as final dose (0.16 mL/m^2^). Five replicates of each treatment were placed throughout the greenhouse experiment. The treatments were in five groups: G1, control tomato plants; G2, treatment with *R. solani* only; G3, plants treated with *Trichoderma*; G4, plants treated with *Trichoderma* and *R. solani*; G5, plants treated with *R. solani* and fungicide. Tomato leaf samples collected 15 days after transplanting from all treated plants in a greenhouse, defense-related genes, and total phenolic compounds in response to treatments were evaluated.

Tomato roots were screened for disease index 30 days after transplantation using a scale of 0–5, according to browning which appeared on the roots of tomato plants [5,35], where 0 = symptomless, 1 = 0–25% browning of the roots, 2 = 26–50% browning of roots, 3 = 51–75% roots browning, 4 = 76–100% browning of roots, 5 indicates the plant has completely died. After observing the tomato plants, the plant disease index (DI) was determined according to the following equation:

$$\mathrm{DI}\;\%=\frac{\left(\sum \mathrm{of}\;\mathrm{all}\;\mathrm{numerical}\;\mathrm{ratings}\right)}{\left(\mathrm{Maximum}\;\mathrm{rate}\;\mathrm{of}\;\mathrm{disease}\times \mathrm{number}\;\mathrm{of}\;\mathrm{all}\;\mathrm{screened}\;\mathrm{plants}\right)}\times 100$$

∑ of all numerical ratings means the sum of all disease ratings; the maximum rate of disease means the maximum disease grade, which in our study is five (the reference scale ranged from 0 to 5); and the number of all screened plants means the total number of ratings in each treatment. Additionally, the measurements of *Trichoderma* effects on the various growth metrics, including plant height and root length, fresh weight of shoot and root system, dry weight of shoot and root systems, as well as the total chlorophyll contents, were tested using SPAD 502 Plus Chlorophyll Meter (Spectrum Technologies Inc., Aurora, IL, USA).

### 2.6. Analysis of Defense-Related-Genes 

#### 2.6.1. Extraction of RNA and Synthesis of cDNA 

Fresh tomato leaves were collected 15 days after transplanting, and 0.1 g of leaves were used for extraction of RNA, using the method of guanidium isothiocyanate (Merck KGaA, Darmstadt, Germany) as described previously [36]. To estimate the purity and concentration of the extracted RNA, Nano SPECTROstar (BMG Labtech, Ortenberg, Germany) was used, while to confirm the RNA integrity, gel electrophoresis agarose was used. Two micrograms of isolated RNA treated with DNase I (Fisher Scientific Inc., Waltham, MA, USA) were used for cDNA synthesis in a reverse transcription reaction (RevertAid First Strand cDNA Synthesis Kit, Catalog no. K1622, Thermo Fisher Scientific Inc., Waltham, MA, USA) [37]. The synthesized cDNA was kept at −80 °C to be used for qRT-PCR. 

#### 2.6.2. Assay of qRT-PCR 

Using the qPCR, the expression level of genes related to pathogenesis (*PAL*, *HQT*, and *CHS*) were examined and normalized using the *β-actin* gene as a reference. Nucleotide sequences of all used primers are presented in Table 1. The experiments were conducted three times for each sample. The qRT-PCR was performed using a Rotor-Gene 6000 QIAGEN (QIAGEN N.V., ABI System, Venlo, The Netherlands) with Thermo SYBR Green Mix (Foster, CA, USA) [38]. Using the 2^−ΔΔCt^ algorism, the levels of relative expression of the studied genes were determined [39].

### 2.7. Oxidative Stress Markers 

All the chemicals, substrates and reagents used in this section and Section 2.8. were purchased from Merck KGaA (Darmstadt, Germany). All the oxidative stress markers and antioxidant enzymes measurments were determined by Novaspec II spectrophotometer (Amersham Pharmacia Biotech Uk Limited, Buckinghamshire, UK) 

#### 2.7.1. Analysis of Malondialdehyde 

Thiobarbituric acid (TBA) was used to evaluate levels of malondialdehyde (MDA), as Heath and Packer [40] described. Tomato leaf samples (0.1 g) were ground in 1 mL of 0.1% trichloroacetic acid (TCA), and the mixture was centrifuged for 30 min at 10,000 rpm. Four mL of TBA solution (0.5% TBA: 20% TCA) was mixed with collected supernatant (1 mL) and maintained for 30 min at 95 °C. The mixture was submerged in ice immediately to stop the reaction, formed color was detected at 600 nm as an indicator of the concentration of malondialdehyde and expressed as µM/g fresh weight (FW).

#### 2.7.2. Determination of Hydrogen Peroxide 

Hydrogen peroxide (H_2_O_2_) was measured in fresh leaf samples of tomatoes using the KI method with a simple modification [41]. Fresh plant samples (100 mg) were crushed using TCA 0.1% and then centrifuged to collect a pure homogenate. The H_2_O_2_ evaluation was measured by mixing 1 mL of plant homogenate with KI solution (2 mL) (1 M KI in 10 mM phosphate buffer, pH 7.0). The absorbance was recorded after 20 min at 390 nm, and the results were reported as µM/g FW utilizing the extinction coefficient of H_2_O_2_ (0.28 M^−1^ cm^−1^).

### 2.8. Antioxidant Enzymatic Activities Measurement

#### 2.8.1. Polyphenol Oxidase Enzyme 

To detect the activity of polyphenol oxidase (PPO), we used the method described by Zhu [42]. Homogenized leaf sample (1 g) was mixed with 250 µL of sodium phosphate buffer (50 mM) (0.1 M, pH 6.5) then centrifuged at 8500 rpm for 30 min at 4 °C. The enzyme extract supernatant (0.1 mL) was mixed with 0.1 mM pyrogallol and 25 mM phosphate buffer (pH 6.8). Pyrogallol was not added to the control mixture. The rate at which absorbency increased at 525 nm was used to calculate the sample’s absorbance. 

#### 2.8.2. Peroxidase Enzyme

Peroxidase (POX) enzyme was measured using 1 g of tomato leaves homogenized and mixed with 5 mL buffer (pH 7.0) with EDTA 0.1% and polyvinylpyrrolidone 10%, then centrifuged at 12,000 rpm for 20 min at 4 °C [43]. The collected supernatant (100 µL) was combined with 0.1 mL of 20 mM guaiacol and 40 µL of 0.1% H_2_O_2_. Absorbance was recorded at 470 nm, and the enzyme activities were expressed as µM/g FW.

#### 2.8.3. Catalase Enzyme

The catalase enzyme (CAT) was estimated by combining 12.5 µL of enzyme extract with 478.5 µL of potassium phosphate buffer (25 mM), which had a final concentration of 10 mM H_2_O_2_ [44]. At 240 nm, catalase activity was recorded and represented as µM/g FW.

#### 2.8.4. Superoxide Dismutase Enzyme

To measure the superoxide dismutase enzyme (SOD), the crude plant extract (0.1 mL) in phosphate buffer (pH 7.0) was mixed with 50 µM nitro blue tetrazolium, 12 mM L-methionine, 0.1 mM EDTA, 10 µM riboflavin, and 50 mM sodium carbonate. The final volume of the reaction was completed to 3 mL by adding 50 mM phosphate buffer (pH 7.6) [45]. The control reaction was without plant extract. To begin the photochemical reaction, the mixtures were introduced to fluorescent lights for 15 min. After that, it was kept in the dark to measure at 560 nm. One unit of SOD activity was defined by a reduction of photochemical (50%) [46]. As µM/g FW, the activity of SOD was expressed.

### 2.9. Polyphenolic Components in Tomato Leaves

#### 2.9.1. Preparation of Tomato Samples for Phenolic Analysis 

Tomato leaf samples were collected 15 days after transplanting and then kept for a week at room temperature to get dry and crushed in a grinder mill to a fine powder (Moulinex AR1044, Paris, France). Two grams of the dry powder were immersed for two days in ethanol 95% (15 mL) [47]. Whatman filter paper No. 1 was used to filter the mixture, and a rotary evaporator was used to evaporate and concentrate the obtained extract in order to completely eliminate the ethanol. The obtained tomato plant extract was reserved at 4 °C in a brown bottle until further analysis. 

#### 2.9.2. HPLC Analysis of Tomato-Collected Leaves Extract

The different polyphenolic components of the tomato sample extract from the treatment of greenhouse experiment were determined using an HPLC analysis with an Agilent 1260 series (Waldbronn, Germany). Eclipse column C18 (4.6 mm × 250 mm i.d., 5 μm) was used for the separation. The mobile phase contains water (A) and trifluoroacetic acid in acetonitrile 0.05% (B) at a 0.9 mL/min flow rate. The following linear gradient was sequentially coded into the mobile phase: 0 min (82% A); 0 to 5 min (80% A); 5 to 8 min (60% A); 8 to 12 min (60% A); 12 to 15 min (82% A); 15 to 16 min (82% A) and 16 to 20 (82% A). Multi-wavelength detector was observed at 280 nm. 5 μL were used for each sample. The column temperature was kept constant at 40 °C.

### 2.10. Statistical Analysis

The data were analyzed using CoStat software, and significant differences were estimated using Tukey’s honest significant differences technique (H.S.D.) at a *p* ≤ 0.05, with standard deviation (SD) presented as a column bar or values. Up-regulation of a gene means that the relative expression levels are greater than 1, whereas down-regulation means values less than 1.

## 3. Results

### 3.1. Rhizoctonia, Trichoderma Isolation and Identification

Five *Rhizoctonia solani* isolates isolated from tomato plant roots with rot symptoms, and the most virulent isolate of *R. solani* R4 was selected based on the pathogenicity test (Table S1). The morphological characteristics of *R. solani* hyphae were found to be septate multinucleate, while conidia and rhizomorphs were never observed. The isolation from tomato rhizospheric soil revealed 12 *Trichoderma* isolates.

### 3.2. Effect of Trichoderma Isolates on R. solani In Vitro

**Figure 1:**
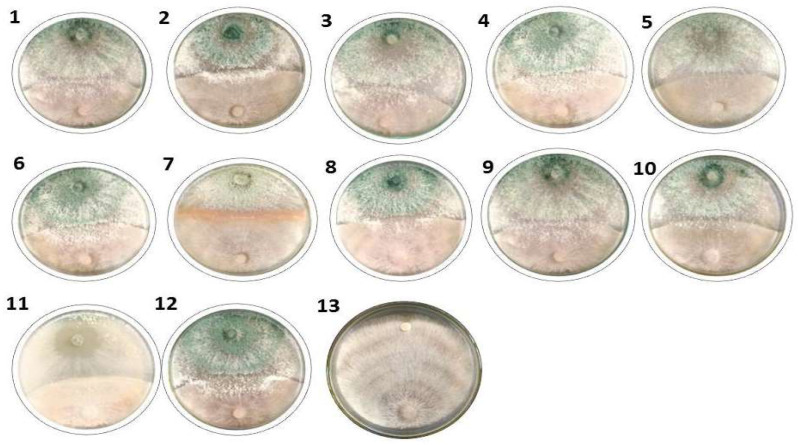
Inhibition effect of *Trichoderma* isolates against *R. solani* growth under laboratory conditions; *Trichoderma* isolates (1–12) and control (13).

The dual culture method was used to test the potential of *Trichoderma* spp. to inhibit *R*. *solani* (R4) growth in vitro. Twelve *Trichoderma* isolates were used to combat root rot pathogen *R. solani* compared to the control (without any treatment) (Table 2 and Figure 1). All tested isolates of *Trichoderma* showed an inhibition effect on *R*. *solani* growth (Table 2), in which *Trichoderma* isolate T3 was significantly the most effective (76.93%), followed by T12 (74.44%) and T9, T11 with inhibition effect 47.04%, while the lowest effect of *Trichoderma* isolates recorded from T4 (61.85%). The antifungal test was used to study the ability of *Trichoderma* isolates to inhibit *R. solani* growth. Among the twelve *Trichoderma* isolates, T3 was most effective against *R*. *solani*, which was selected for further study under greenhouse conditions.

### 3.3. Effect of Fungicides on R. solani In Vitro

**Figure 2:**
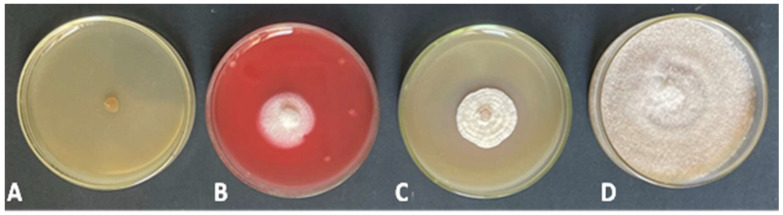
Response of *R. solani* isolate to different fungicides at 1 ppm concentration. The fungicides were Uniform^®^ (**A**), Hattrick^®^ (**B**), and Rizolex^®^ (**C**) compared with control (**D**).

All the tested fungicides demonstrated variable degrees of control over the pathogen radial mycelial growth and significantly differed from the control (Figure 2). Uniform^®^, Rizolex^®^, and Hattrick^®^ fungicides were effective against *R. solani* by inhibiting the growth of fungal mycelium. At the concentration (1 ppm), Uniform^®^ completely inhibited the radial mycelial growth of *R. solani* (100%), while Hattrick^®^ and Rizolex^®^ fungicides inhibition percentages were 64.07 and 62.96%, respectively, compared with the control. For that, we selected Uniform^®^ fungicide for further study under greenhouse conditions.

### 3.4. Molecular Identification of R. solani and Trichoderma *spp.*

*Rhizoctonia solani* was identified at a molecular level using ITS1/ITS4 primers. Molecular results confirmed the primary identification of the evaluated isolate in this study. The identified isolate *R. solani* strain R11 was deposited in the NCBI-GenBank database under accession no. OP456528. Comparing ITS nucleotide sequence with *R. solani* isolates in the NCBI-GenBank database showed that the highest homogeneity was 100% with *R. solani* isolate from tomato (HG934419).

**Figure 3:**
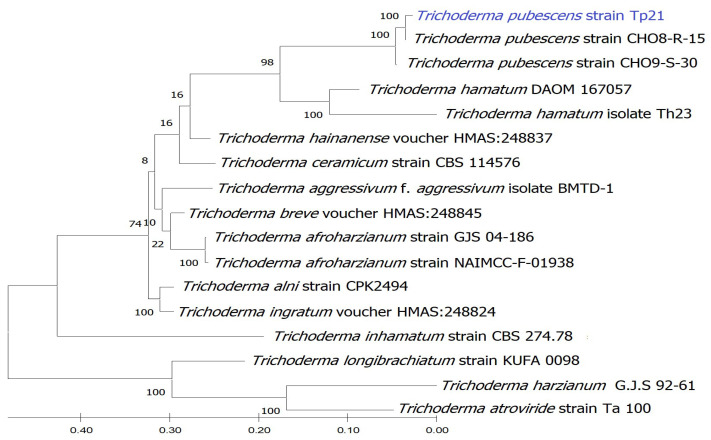
Cladogram of *Trichoderma* spp. sequences aligned with *T. pubescens* strain Tp21 upon partial sequences of ITS, *tef*-1, and *rpb*2 according to the maximum likelihood method.

On the other hand, ITS region, *tef*-1, and *rpb*2 genes were used for identifying the most effective isolate of *Trichoderma* in inhibiting *R. solani* fungus. Using NCBI-BLAST alignment, *Trichoderma* isolate was highly similar to *Trichoderma pubescens*. Phylogenetic analysis was performed using three molecular markers ITS (OP456527), *tef*-1 (OP491464), and *rpb*2 (OP491463) datasets to describe species limits. Bootstrap 1000 subgroups indicated the importance of each branch in the alignment (only values higher than 24% are displayed). A multiple sequence alignment in the maximum likelihood method using Mega X revealed the relationship of almost all *Trichoderma* spp., reference isolates could be clearly distinguished on the level of species and divided into various clusters and clades (Figure 3).

### 3.5. Effect of T. pubescens on R. solani Root Rot In Vivo 

Under greenhouse conditions, *T. pubescens* was tested for its activity against *R. solani,* causing root rot in tomatoes. The severity of the root browning symptoms for each treatment (on a scale from 0 to 5) was used to record the disease index (DI%) in Table 3. *T. pubescens* treatment significantly reduced the DI% compared with control plants (G1). Applying *T. pubescens* on tomato plants in G4 treatment showed a significant reduction in plant disease index (16.00%) compared with G5 treatment (14.67%). Meanwhile, DI% was 78.67% in the G2 treatment compared to the G1 and G3 treatments (0.0%).

### 3.6. Efficacy of T. pubescens on Tomato Growth Parameters

In the experiment conducted in a greenhouse, *T. pubescens* treatment demonstrated a significant increase (*p* ≤ 0.05) in the growth parameters of treated plants (Table 4). Additionally, the *T. pubescens* treatment significantly impacted plant height. Plant height was recorded as 21.27 cm in *T. pubescens* treatment (G3), followed by *T. pubescens* + *R. solani* (G4), which recorded 19.23 cm. Compared to the *R. solani* treatment (G2, 11.10 cm) and *R. solani* + fungicide treatment (G5, 13.27 cm), G3 and G4 treatments significantly increased root length by 10.37 and 9.23 cm, respectively (Table 4). *T. pubescens* treatments alone (G3) or when applied in inoculated plants (G4) increased the fresh weight of the shoot (6.10 and 5.38g, respectively) and root-fresh weight (3.40 and 3.23 g, respectively) compared to the control treatment (G1). Tomato roots’ dry weight was changed after being treated with *T. pubescens* (G3) and fungicide treatment (G5) compared with G2 and G1 treatments (Table 4). *T. pubescens* treatment (G3) effectively increased the chlorophyll content (37.70 SPAD value), followed by *T. pubescens* + *R. solani* treatment (G4), with a SPAD value of 36.70. In contrast, *R. solani* + fungicide treatment (G5) showed a SPAD value of 35.10 compared to the control (G1, 35.27), and plants inoculated with *R. solani* (G2) showed a SPAD value of 23.63 (Table 3).

### 3.7. Defense-Related Enzymes Activity 

**Figure 4:**
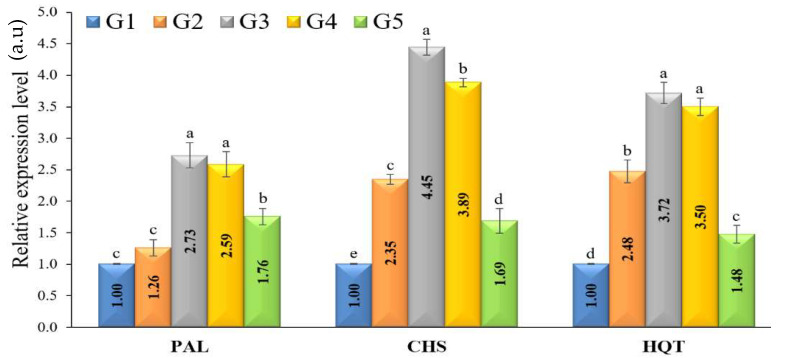
A relative expression values of the *PAL*, *CHS,* and *HQT* genes at 15 days after transplanting in different treatments. a.u: arbitrary units. Significant differences are represented by the various letters (a–e) at *p*-value ≤ 0.05. G1 = untreated tomato plants (control), G2 = plants inoculated by *R. solani* only, G3 = plants treated with *Trichoderma pubescens*, G4 = plants inoculated by *R. solani* and treated with *T. pubescens* and, G5 = plants inoculated by *R. solani* and treated with Uniform^®^ fungicide.

At 15 days after transplanting, the expression levels of three defense-related-genes (*PAL*, *CHS*, and *HQT*) have increased significantly in plants treated with *T. pubescens* in comparison with the untreated plants (*p* ≤ 0.05). All treatments significantly upregulated *PAL* as compared to the control group (G1) (Figure 4). *T. pubescens* treatment (G3) showed the highest level of relative expression (2.729-fold higher than the control), followed by G4 (*T. pubescens* + *R. solani*) and G5 (*R. solani* + fungicide) treatments with expression levels 2.586- and 1.757-fold, respectively. Like *CHS*, plants treated with *T. pubescens* showed upregulated *CHS* expression levels. The highest expression level (4.447-fold) was observed in G3, followed by G4 (3.887-fold higher) and G2 (2.347-fold higher). A significant increase in *HQT* expression was observed in all treatment groups compared to the control, particularly in plants treated with *T. pubescens* (Figure 4). The most considerable transcriptional level (3.72-fold) was recorded in the G3 treatment, followed by G4, G2, and G5, with expression levels 3.503-, 2.477-, and 1.477-fold higher than the control, respectively.

### 3.8. H_2_O_2_ and MDA Kinetics 

**Figure 5:**
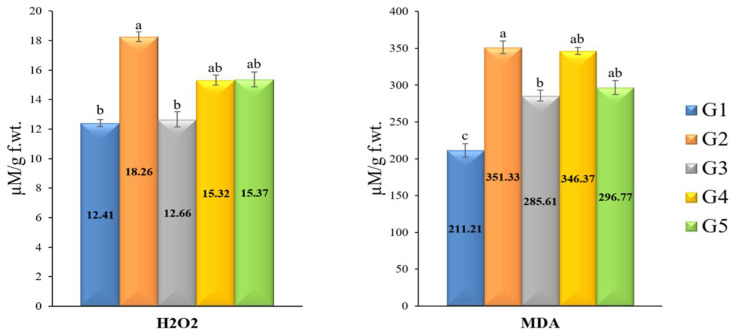
Estimated oxidative stress markers for H_2_O_2_ and MDA in tomato plants under greenhouse conditions. Significant differences are represented by the various letters (a–c) at *p*-value ≤ 0.05. G1 = untreated tomato plants (control), G2 = plants inoculated by *R. solani* only, G3 = plants treated with *Trichoderma pubescens*, G4 = plants inoculated by *R. solani* and treated with *T. pubescens* and, G5 = plants inoculated by *R. solani* and treated with Uniform^®^ fungicide.

Two oxidative stress markers (H_2_O_2_ and MDA) were estimated. The results for H_2_O_2_ showed that plants inoculated with *R. solani* showed the highest level (18.26 µM/g FW), followed by *R. solani*-inoculated plants treated with fungicide (15.37 µM/g FW) compared with untreated plants (12.41 µM/g FW). However, the tomato plants from the two treatments, *T. pubescens* (G3) and *T. pubescens* + *R. solani* (G4), showed a reduction in H_2_O_2_ content compared to the *R. solani* treatment (G2). *T. pubescens* treatment (G2) recorded 12.66 µM/g FW, while *T. pubescens* + *R. solani* (G4) showed 15.32 µM/g FW (Figure 5). Similar to H_2_O_2_, MDA was elevated upon *R. solani* infection. The treatment of *R. solani* + fungicide (G5) and *R. solani* alone (G2) showed the greatest H_2_O_2_ level (351.33 and 346.37 µM/g FW, respectively) compared to the untreated control, which showed a significant increase (211.21 µM/g FW). At the same time, plants treated with *T. pubescens* + *R. solani* (G4) recorded 296.77 and 285.61 µM/g FW for G3 treatment (*T. pubescens* alone) with significant decreases in the MDA.

### 3.9. Antioxidant Enzymes Activities

**Figure 6:**
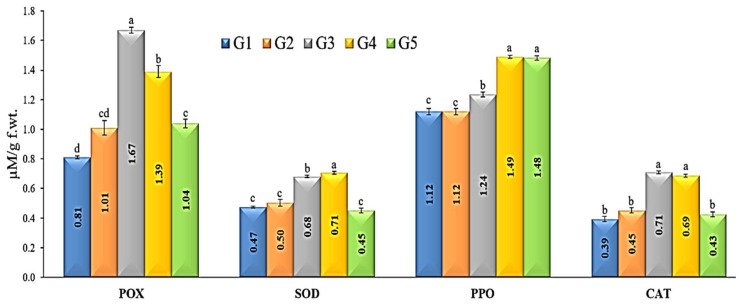
Activities of antioxidant enzymes in tomato plants with *T. pubescens* and inoculated with *R. solani*. Significant differences are represented by the various letters (a–d) at *p*-value ≤ 0.05. G1 = untreated tomato plants (control), G2 = plants inoculated by *R. solani* only, G3 = plants treated with *Trichoderma pubescens*, G4 = plants inoculated by *R. solani* and treated with *T. pubescens* and, G5 = plants inoculated by *R. solani* and treated with Uniform^®^ fungicide.

Four antioxidant enzymes, namely SOD, PPO, POX, and CAT, were distinguished upon *R. solani* infection and *T. pubescens* as well as fungicide treatment (Figure 6). *T. pubescens* isolate induced the antioxidant defense system and enhanced the four-enzyme content significantly in tomato plants, as recorded in group treatments G3 and G4. The *T. pubescens* + *R. solani* treatment (G4) exhibited the highest PPO activity value (1.49 µM/g FW), followed by *R. solani*-inoculated plants treated with fungicide (G5, 1.482 µM/g FW). At the same time, treatment with *T. pubescens* alone (G3) induced the PPO activity by 1.236 µM/g FW. Peroxidase (POX) activity was elevated in response to *R. solani* infection in plants treated with *T. pubescens* (Figure 6). Compared with control (G1), infected plants treated with *T. pubescens* (G4) exhibited the highest level of peroxidase activity, followed by plants treated with *T. pubescens* (G3). Furthermore, *R. solani* inoculated plants treated with fungicide (G5) displayed a slight increase in peroxidase activity more than the control. Regarding antioxidant enzyme CAT activity, G3 and G4 treatments showed the greatest level of content (0.708 and 0.685 µM/g FW, respectively) compared with the G5 treatment (0.425 µM/g FW). In addition, G2 treatment exhibited 0.452 µM/g FW activity compared to G1 plants (0.393 µM/g FW). Concerning the activity of superoxide dismutase (SOD), results revealed that SOD was significantly increased upon *T. pubescens* treatment with or without *R. solani* (Figure 6). The G4 treatment showed the highest SOD activity, followed by G3 treatment (0.705, 0.680 µM/g FW, respectively). In comparison, the lowest value for SOD activity was obtained for G1 plants (0.474 µM/g FW).

### 3.10. Phytochemical Analysis of Tomato Leaf Extract 

**Figure 7:**
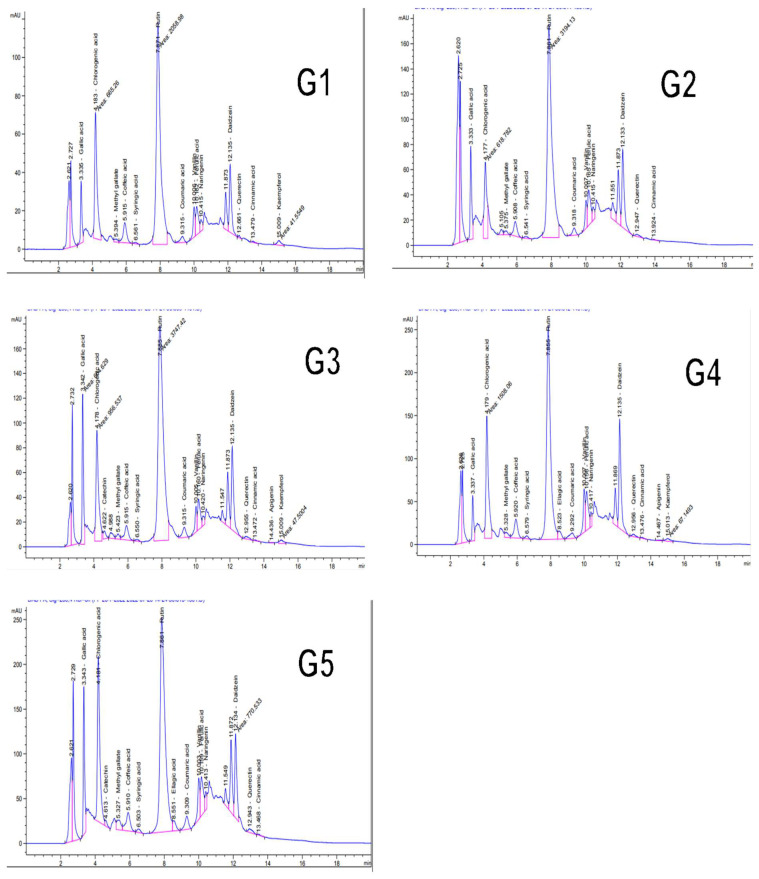
Identified polyphenolic substances by HPLC chromatography in ethanol extract of tomato leaves 15 days after transplanting of various treatments. G1 = untreated plants (Control), G2 = plants inoculated with *R. solani*, G3 = treatment with *T. pubescens*, G4 = plants inoculated with *R. solani* and *T. pubescens* and, G5 = plants inoculated with *R. solani* and Uniform^®^ fungicide.

The HPLC chromatograms for ethanolic extracts for different groups of treatments G1, G2, G3, G4, and G5 tomato plants were presented in Figure 7. HPLC analysis showed that the total contents of 19 polyphenolic compounds were 35,205.91, 35,591.70, 56,032.64, 43,031.03, and 41,038.68 µg/g for G1, G2, G3, G4, and G5, respectively (Figure 7). The major detected compounds were gallic acid, chlorogenic acid, ferulic acid, methyl gallate, caffeic acid, syringic acid, pyrocatechol, ellagic acid, coumaric acid, and cinnamic acid, while flavonoid compounds were vanillin, catechin, rutin, quercetin, naringenin, daidzein, quercetin, apigenin, and kaempferol. The most prevalent phenolic compounds (µg/g) were chlorogenic acid (7875.70, 5158.17, 9981.89, 9613.27, and 9282.39), gallic acid (1367.53, 2294.84, 4254.70, 1275.36, and 2776.91), ferulic acid (1136.84, 1581.69, 1619.09, 9613.27, and 1473.40), caffeic acid (1235.52, 903.82, 1319.00, 1430.01, and 1231.44) in G1, G2, G3, G4, and G5 extracts, respectively. On the other hand, naringenin as a flavonoid compound was overexpressed in G2 treatment with an accumulation value (606.62 µg/g) compared to G1 plants (500.20 µg/g) (Table 5).

## 4. Discussion

Many fields are frequently affected by the *Rhizoctonia solani* fungus disease, which significantly negatively impacts seed germination and growth of many plants and causes root rot diseases [5,48]. Fungicides, as chemical controls, are regularly used and effectively manage *R. solani* fungus. However, using chemicals to control fungal infections in plants might not always be acceptable. Chemical control of plant diseases is becoming less popular because of the risks that come with it [23]. As biocontrol agents, many microorganisms are used because they are a safe alternative to harmful fungicides and are sustainable and good for the environment [49,50]. *Trichoderma* spp. are known to be very effective antagonist fungi that have biocontrol activity on many other fungi, such as *R. solani*, *Verticillium dahliae*, and *Sclerotium rolfsii* [51]. 

The morphological characteristics of the genus *Rhizoctonia*, an anamorphic mycelial septate fungus without asexual spores, were consistent in identifying *R. solani* isolated from tomato plants [5]. The isolated fungus from tomato root rot was identified morphologically and molecularly as *R. solani*. For molecular identification of *Trichoderma*, ITS, *tef*-1, and *rpb2* primers were used as multigenic analysis and were characterized as *T. pubescens.* Also, to test the ability of *T. pubescens* to suppress *R. solani* and enhance the tomato plants’ growth parameters, we performed an experiment composed of the following treatments: G1, untreated tomato plants (control); G2, plants inoculated by *R. solani* only; G3, plants treated with *T. pubescens*; G4, plants inoculated by *R. solani* and treated with *T. pubescens*; and, G5, plants inoculated by *R. solani* and treated with Uniform^®^ fungicide.

Our results showed the ability of *T. pubescens* to inhibit *R. solani* growth by up to 76.39%, as *T. pubescens* grows faster than *R. solani* under laboratory conditions. Siameto et al. [52] studied the growth inhibition of *R. solani* using *T. harzianum* and found that the highest inhibition percentage of *R. solani* inhibition was 61.55%, while the lowest value was 25.88%. Guedez et al. [53] found that the growth-inhibitory activity of different isolates of *T. harzianum* against *R. solani* ranged from 62.0 to 72.0%. According to Ramrez-Cario et al. [54], *Trichoderma* species have a significant advantage over the pathogens *Alternaria alternata* and *Fusarium oxysporum* in the competition for nutrients and growth area. This is due to their fast rate of growth and development. Competition is a mechanism that occurs when there is a lack of nutrients and space for living. *T. pubescens* was evaluated for its effect on *R. solani* under greenhouse conditions and revealed that the severity of the disease symptoms was significantly reduced. The most frequent reason for microbial death is nutrition deficiency. A vital method of preventing plant diseases is the use of biological controls to contend for scarce nutrition [55].

*T. pubescens* increased the length of the shoot and root systems, as well as the fresh and dry weight of the shoot and root systems and the amount of chlorophyll compared to control plants (G1) and plants that were infected by *R. solani* (G2). According to our findings, tomato plants treated with *T. pubescens* were able to suppress *R. solani* in the G4 treatment. These results align with what Harman et al. [56] found about the improvement in the growth of shoot and root systems. The acquired statistics are also consistent with the findings of Yedidia et al. [57], who claimed that *T. pubescens* treatment had a higher impact on cucumber plants than control plants, increasing the root length by 75%, shoot length by 95%, and dry weight by 80% [57]. The amount of chlorophyll in melon plants that were treated with *T. harzianum* went up [58]. 

Tomato plants treated with *T. gamsii* resulted in similar findings of improved plant growth [59]. The secretion of auxins, gibberellins, and cytokinins may be responsible for increased plant growth. Their siderophore or antibiotic synthesis and direct or indirect stimulation of nutrient uptake may also contribute to the rise in biomatter production [59]. *Trichoderma*-secreted compounds are produced to protect plants from harmful rhizosphere organisms. *Trichoderma* can help plants grow by increasing soil nutrient uptake, speeding up photosynthesis, and enhancing growth parameters. This is possible because many microorganisms can produce indole-acetic acid (IAA) [60]. In addition to being crucial for root hair and lateral root development, the indole acetic acid hormone is also thought to be a major regulator for plant root and shoot growth. *Trichoderma* species from different regions can release IAA and promote the development of plants like tomato and cucumber [16]. *Trichoderma* spp. can secrete secondary metabolites like 6-n-pentyl-6H-pyran-2-one (6PP), harziandione, gliotoxin, viridin, harzianopyridone, and peptaibols, which have a significant effect as growth promoters [56]. 

In our study, *T. pubescens* effects on relative expressions of three defense-related genes (*PAL*, *CHS*, and *HQT*) were determined 15 days after transplanting. As mentioned before, in plants infected with *R. solani*, SAR was triggered, while in *Trichoderma* treatments, an ISR mechanism was induced [22]. Different secondary metabolites activate the expression of PR proteins through the interaction between *Trichoderma* and tomato plants, which triggers defense mechanisms against plant pathogens [5]. The effect of *T. pubescens* against *R. solani* is due to growth enhancement, at least partly. Many enzyme activities were involved in the interaction among the *Trichoderma*-plant-pathogen dilemma. The enzyme SOD has a role in the early defensive reactions. It is classified as the first detoxification phase. Superoxide anion dismutation into hydrogen peroxide is catalyzed by SOD [61]. Zehra et al. [62] stated that *T. harzianum* showed an increase in the activity of SOD in infected tomato plants with *F. oxysporum*. In our study, compared to all treatments, only plants that were treated with *T. pubescens* (G3) had a small amount of H_2_O_2_ built up in their leaves. In line with Zehra et al. [62], MDA concentrations increased in the tomato plants that were infected with *R. solani* (G2) or in combination with *T*. *pubescens* (G4) during our investigation. When *Trichoderma* was used to treat tomato plants (G3), the MDA levels were much lower than when plants were exposed to pathogens (G2). Our results revealed that *T. pubescens* improved defense and detoxification systems, which cause rapid and effective responses to pathogen inoculation. Also, when tomato is stressed by fungi, the activities of flavonoids, phenolics, PPO, CAT, and SOD may be very important for their survival. CAT is known to protect plant cells under stress exposure from ROS oxidative damage by converting ROS components into less toxic and more stable molecules like oxygen and water [63]. In our experiment, the CAT enzyme was significantly raised in plants treated with *T*. *pubescens* (G3) and *T*. *pubescens* + *R. solani* (G4). Masuta et al. [64] discussed that CAT might make cell walls more resistant, turn on defense genes, and increase the signal of SA.

Extracted tomato plants’ antibacterial and antifungal activities are linked to their chemical composition and the functional groups of the significant compounds (flavonoids, phenols, terpenes, chlorogenic acid, and caffeic acid) [65,66]. Many substances produced by plants, such as phenolic acids, polyphenols, flavonoids, and terpenoids, have been characterized as effective against various pathogenic microbes. HPLC analysis of tomato leaf extracts in all treatments revealed an increase in polyphenolic compound content in plants treated with *T. pubescens* alone (G3) or in combination with *R. solani* (G4) compared to control plants (G1) with values 56,032.64 µg/g and 43,031.03 µg/g, respectively. The main detected phenolic compounds were gallic acid, chlorogenic acid, caffeic acid, syringic acid, ellagic acid, coumaric acid, and cinnamic acid. In contrast, the most prevalent flavonoid compounds were catechin, rutin, naringenin, and kaempferol [67,68]. Also, tomato leaves have more flavonoids, solavetivone, lubimin, phytuberin, phytuberol, rishitin, and glutinosone. These chemicals have toxic antimicrobial properties, so they protect the plant from a wide range of pathogens and pests. Because of this, the phenolics that build up in plants treated with *Trichoderma* can act as electron and hydrogen donors, protecting root tissue from damage caused by oxidation when pathogens attack. The analysis of tomato leaf extracts using HPLC is consistent with tomatoes protected from the *R. solani* infection as detected in onions by Ortega-Garca et al. [69].

## 5. Conclusions

Our investigation studied the effect of *T. pubescens* on tomato root rot disease caused by *R. solani*. The study revealed that *T. pubescens* effectively controls *R. solani* in vitro and in vivo. A substantial increase in root length, plant height, shoot fresh and dry, and root fresh and dry weight was indicated after applying *T. pubescens* to tomato plants in vivo. The treatment with *T. pubescens* exhibited a lower disease index than the control. At 15 days after inoculation, promising increases in the relative expression levels of three defense-related genes (*PAL, CHS,* and *HQT*) were observed in all *T. pubescens* treated plants. *T. pubescens* G3 *and* G4 treatments exhibited increasing antioxidant enzyme production (POX, SOD, PPO, and CAT), and high MDA and H_2_O_2_ levels were observed in the infected plants. The HPLC results of *T. pubescens* G3 and G4 treatments showed increasing in chlorogenic and coumaric acids. Therefore, the ability of *T. pubescens* to inhibit the growth of *R. solani*, enhance the development of tomato plants, and induce systemic resistance supports the application of *T. pubescens* as a potential bioagent for managing root rot disease and productivity increase of crops.

## Figures and Tables

**Table 1:** List of sequences of primer nucleotide used in this study.

Gene	Amplified Region	Primer Direction	Sequence (5’-3’)	References
ITS	Internal transcribed spacer	ITS1	TCCGTAGGTGAACCTGCGG	[30]
		ITS4	TCCTCCGCTTATTGATATGC	
*Rpb2*	RNA polymerase II subunit 2	fRPB2-5f	GAYGAYMGWGATCAYTTYGG	[31]
		fRPB2-7cr	CCCATRGCTTGYTTRCCCAT	
*Tef*-1	Translation elongation factor 1 alpha	EF1-728F	CATCGAGAAGTTCGAGAAGG	[32]
		TEF1LLErev	AACTTGCAGGCAATGTGG	
*PAL*	Phenylalanine ammonia-lyase	PAL-f	ACGGGTTGCCATCTAATCTGACA	[33]
		PAL-r	CGAGCAATAAGAAGCCATCGCAAT	
*HQT*	Hydroxycinnamoyl Co A quinate hydroxycinnamoyl transferase	HQT-f	CCCAATGGCTGGAAGATTAGCTA	[34]
		HQT-r	CATGAATCACTTTCAGCCTCAACAA	
*CHS*	Chalcone synthase	CHS-f	CACCGTGGAGGAGTATCGTAAGGC	[34]
		CHS-r	TGATCAACACAGTTGGAAGGCG	

**Table 2:** Efficacy of *Trichoderma* isolates on *R. solani* growth compared with control in vitro.

Treatments	Inhibition % ± SD *
T1	70.74 ± 2.57 c
T2	68.52 ± 3.39 c
T3	76.93 ± 3.21 a
T4	61.85 ± 4.49 d
T5	68.52 ± 3.21 c
T6	68.52 ± 3.21 c
T7	70.37 ± 3.21 c
T8	62.96 ± 3.21 d
T9	74.07 ± 3.21 b
T10	69.26 ± 0.64 c
T11	74.07 ± 1.70 b
T12	74.44 ± 2.94 b
Control	00.00 ± 0.00 e

* SD means standard deviation. Different letters alongside the inhibition % data values mean the values were significantly different at *p*-value ≤ 0.05.

**Table 3:** Effect of *Trichoderma pubescens* on *Rhizoctonia solani* disease index (DI%) and chlorophyll content on tomato plants in vivo.

Treatment	Disease Index ± SD *	Total Chlorophyll Content (SPAD) ± SD
G1	00.00 ± 0.00 c	35.27 ± 0.90 b
G2	78.67 ± 5.58 a	23.63 ± 1.07 c
G3	00.00 ± 0.00 c	37.70 ± 0.70 a
G4	16.00 ± 3.65 b	36.70 ± 0.79 ab
G5	14.67 ± 5.54 b	35.10 ± 1.0 ab

* SD means standard deviation. Different letters alongside data values in each column mean the values differed significantly at *p*-value ≤ 0.05. G1 = untreated tomato plants (control), G2 = plants inoculated by *R. solani* only, G3 = plants treated with *Trichoderma pubescens*; G4 = plants inoculated by *R. solani* and treated with *T. pubescens* and, G5 = plants inoculated by *R. solani* and treated with Uniform^®^ fungicide.

**Table 4:** Effect of various treatments on tomato plants growth parameters under greenhouse conditions.

Treatments **	Length (cm) ± SD *		Fresh Weight (g) ± SD		Dry Weight (g) ± SD	
	Shoot	Root	Shoot	Root	Shoot	Root
G1	13.30 ± 0.79 b	06.23 ± 0.06 d	4.40 ± 0.10 b	2.03 ± 0.32 b	1.87 ± 0.15 ab	0.93 ± 0.15 b
G2	11.10 ± 1.11 b	04.93 ± 0.38 e	2.63 ± 0.15 c	1.10 ± 0.20 b	1.57 ± 0.31 b	0.40 ± 0.10 c
G3	21.27 ± 0.64 a	10.37 ± 0.12 a	6.10 ± 0.53 a	3.40 ± 0.10 a	2.47 ± 0.25 a	1.47 ± 0.15 a
G4	19.23 ± 1.44 a	09.23 ± 0.49 b	5.38 ± 0.13 a	3.23 ± 0.12 a	2.37 ± 0.55 ab	1.37 ± 0.06 a
G5	13.27 ± 0.40 b	07.07 ± 0.21 c	4.53 ± 0.32 b	1.70 ± 0.10 b	1.90 ± 0.10 ab	0.83 ± 0.23 b

* SD means standard deviation. Different letters alongside data values in each column mean the values differed significantly at the *p*-value ≤ 0.05. ** G1 = untreated tomato plants (control), G2 = plants inoculated by *R. solani* only, G3 = plants treated with *Trichoderma pubescens*, G4 = plants inoculated by *R. solani* and treated with *T. pubescens* and, G5 = plants inoculated by *R. solani* and treated with Uniform^®^ fungicide.

**Table 5:** Polyphenolic compounds in ethanolic tomato leaf extracts using HPLC analysis.

Compound	Concentration (µg/g)				
	G1 *	G2	G3	G4	G5
Gallic acid	1367.53	2294.84	4254.70	1275.36	2776.91
Chlorogenic acid	7875.70	5158.17	9981.89	9613.27	9282.39
Ferulic acid	1136.84	1581.69	1619.09	1839.91	1473.40
Methyl gallate	133.15	168.65	282.46	291.74	489.70
Coffeic acid	1235.52	903.82	1319.00	1430.01	1231.44
Syringic acid	81.76	126.33	152.03	217.20	204.89
Ellagic acid	0.00	0.00	0.00	1190.75	1083.84
Coumaric acid	136.32	143.42	294.12	173.35	237.75
Cinnamic acid	17.00	9.22	15.46	17.07	11.73
Vanillin	619.55	586.14	709.76	1071.24	805.74
Catechin	0.00	0.00	1251.23	0.00	538.17
Rutin	19,975.11	21,819.77	32,046.74	21,580.30	20,416.05
Naringenin	500.19	606.62	598.33	598.06	548.08
Daidzein	1546.99	2003.50	2671.25	3040.66	1650.84
Querectin	68.89	189.53	298.64	211.84	287.75
Apigenin	0.00	0.00	22.68	35.33	0.00
Kaempferol	511.38	0.00	515.26	444.95	0.00
Total	35,205.91	35,591.70	56,032.64	43,031.03	41,038.68

* G1 = untreated plants (Control), G2 = plants inoculated with *R. solani*, G3 = treatment with *T. pubescens*, G4 = plants inoculated with *R. solani* and *T. pubescens* and, G5 = plants inoculated with *R. solani* and Uniform^®^ fungicide.

## Data Availability

Not applicable.

## Supplementary Materials

The following supporting information can be downloaded at: https://www.mdpi.com/article/10.3390/jof9020167/s1, Table S1: Response of tomato plants to pathogenicity of *Rhizoctonia solani* isolates recorded as disease index (DI%).
