# Supplementary material for: Trichoderma pubescens Elicit Induced Systemic Resistance in Tomato Challenged by Rhizoctonia solani
Source: J Fungi (Basel). 2023 Jan 27;9(2):167. doi: 10.3390/jof9020167 (PMC9961125; doi:10.3390/jof9020167)
Supplement: Supplementary file 1 [file jof-09-00167-s001.zip › jof-2154372-supplementary.pdf]

Table S1. Response of tomato plants to pathogenicity of *Rhizoctonia solani* isolates recorded as disease index (DI%).

**Isolates**	**Disease index ± SD***
R1	41.49 ± 2.26 d
R2	68.16 ± 3.42 b
R3	74.80 ± 3.73 b
R4	83.68 ± 4.36 a
R5	54.26 ± 1.49 c

*SD means standard deviation. Different letters alongside data values in each column mean the values differed significantly at p-value ≤ 0.05.
